# The response of an arboreal mammal to livestock grazing is habitat dependant

**DOI:** 10.1038/s41598-017-17829-6

**Published:** 2017-12-12

**Authors:** Heather Neilly, Lin Schwarzkopf

**Affiliations:** 1 James Cook Drive, Townsville, QLD 4812 Australia; 2 Centre Tropical Biodiversity and Climate Change, James Cook University, Townsville, Australia

## Abstract

Inappropriate livestock grazing is implicated in the decline of vertebrate fauna species globally. Faunal responses to grazing can interact with the vegetation community in which they occur. We measured the response of an arboreal marsupial, the common brushtail possum (*Trichosurus vulpecula vulpecula*) to different cattle grazing strategies and vegetation types, and examined whether micro-habitat selection is driving this response. We hypothesised that where arboreal habitat is intact, brushtail possums would be resistant to the impacts of heavy grazing. We conducted a mark-recapture survey among four grazing treatments and in two vegetation types (Box and Ironbark), at a 20-year grazing trial in northern Australia. We found that brushtail possums were resistant to the impact of heavy grazing in both vegetation types, but preferred the heavy grazing treatment in the Box vegetation type. Complex arboreal habitat and low ground cover was preferred, and high grass cover and low tree species richness avoided. Most individuals exclusively used one vegetation type, with few using both, suggesting a ‘matrix’ vegetation between the Box and Ironbark may be creating a movement barrier. Vegetation type should provide a context for determining the benefits to arboreal wildlife of adopting a particular grazing management strategy.

## Introduction

The impact of livestock grazing on biodiversity is important, not only due to the vast extent of global rangelands, but the diversity of biomes in which grazing occurs^1^. Grazing alters ground-level structures directly, e.g. by soil compaction, the removal of vegetation and the addition of nutrients^2^. The indirect effects of grazing may further impact lower habitat strata, but can also negatively impact arboreal structures^3^.

While vegetation responses to grazing have been studied in detail, the response of the vertebrate fauna that use these habitats is complex^4^. Vertebrate fauna can increase in abundance with increasing grazing disturbance, decrease in abundance, or remain unchanged^5–7^. These responses depend on the way that grazing alters micro-habitat and the specific habitat requirements of fauna species^8^. Vegetation type, the use of fire, the introduction of non-native grasses and tree clearing, can interact with species’ responses to grazing, often exacerbating negative impacts^9–12^. The complexity and species-specific responses of vertebrates in rangelands, can make it difficult to translate research results into clear management recommendations.

Although grazing is broadly considered a factor contributing to vertebrate species’ declines^13^, arboreal species may be somewhat resistant to grazing disturbance, because they can mostly avoid ground-level impacts, as seen in birds^14,15^ and arboreal reptiles^3^. Arboreal mammals are present in agroecosystems globally, but mammal community grazing studies usually focus on small terrestrial mammals^11,12,16^, with arboreal mammals receiving less attention (but see^17–21^). Due to their prevalence on rangelands, the response of arboreal mammals to grazing needs further attention, so that these species can be considered when devising grazing management strategies.

The common brushtail possum (*Trichosurus vulpecula vulpecula* Kerr 1792, henceforth ‘brushtail possum’), is a medium-sized, arboreal marsupial found in a range of disturbed environments, including tropical savanna rangelands grazed by cattle^22^. They thrive in Australian urban areas, are considered a pest in some agricultural areas in Australia, and are a serious introduced pest species in New Zealand^23–26^. Their adaptability is due to their use of a variety of habitat types, their ability to breed continually and to exploit a variety of seasonal food sources^23,27–29^.

Aside from urban areas, brushtail possum populations have declined in many parts of their native range, most markedly in arid central Australia^23,25,30,31^. These declines are attributed to habitat loss, drought, introduced feral predators and hunting^27,28^. Consequently, reintroduction programs have been successful in areas where feral cats and foxes have been excluded^32^. Whilst brushtail possums are an arboreal species heavily utilising trees, they use rock-holes, caves and burrows for nesting and also frequently walk along the ground to move between trees in areas where the tree canopy is not connected^23,27^. The ability of brushtail possums to persist in disturbed areas if vertical structures are present^33^, combined with their use of the ground to move between trees, suggest that they may benefit from cattle grazing disturbance, where trees are retained and grass cover is reduced. Since most of the brushtail possum range coincides with rangelands, it is important to understand their response to grazing, and investigate if micro-habitat selection is driving this response.

We examined population dynamics, habitat selection and movement of brushtail possum individuals, in response to four different grazing strategies (all without any associated tree clearing), and between two vegetation types, using mark-recapture data. This study took place on a long-term cattle-grazing trial in tropical savanna woodland in northern Australia. We aimed to identify if brushtail possum abundance was influenced by different grazing strategies and vegetation types, and then if individuals were selecting or avoiding certain micro-habitat features. Due to this species’ adaptability to disturbance and the lack of tree clearing associated with our grazing treatments, we expected brushtail possums to be resistant to the impact of heavy grazing. As such, we predicted that brushtail possums would select ground micro-habitat features that were consistent with relatively disturbed areas, but select more complex arboreal micro-habitats.

## Materials and Methods

### Study location

Surveys were conducted at the Wambiana Grazing Trial, southwest of Charters Towers, Queensland, Australia. The 1040 ha trial was established in 1997 by the Queensland Department of Agriculture and Fisheries, to test the impact of different grazing strategies on cattle production^34–36^. At this location there is summer wet season and winter dry season, with an average annual rainfall of 643 mm. Eight paddocks were randomly assigned one of four grazing treatments, therefore each treatment paddock was replicated twice. The grazing treatments are typical management practices in the region: 1) Heavy stocking rate −4–6 ha/Adult Equivalent (AE, defined as 450 kg steer); 2) Moderate stocking rate −8–10 ha/AE; 3) Variable stocking rate – stocking rates adjusted annually based on the end of wet season feed availability, range 3–12 ha/AE and; 4) Rotational Wet Season Spelling – a third of the paddock spelled each wet season 7–10 ha/AE (see^35^ for detailed treatment descriptions).

**Figure 1:**
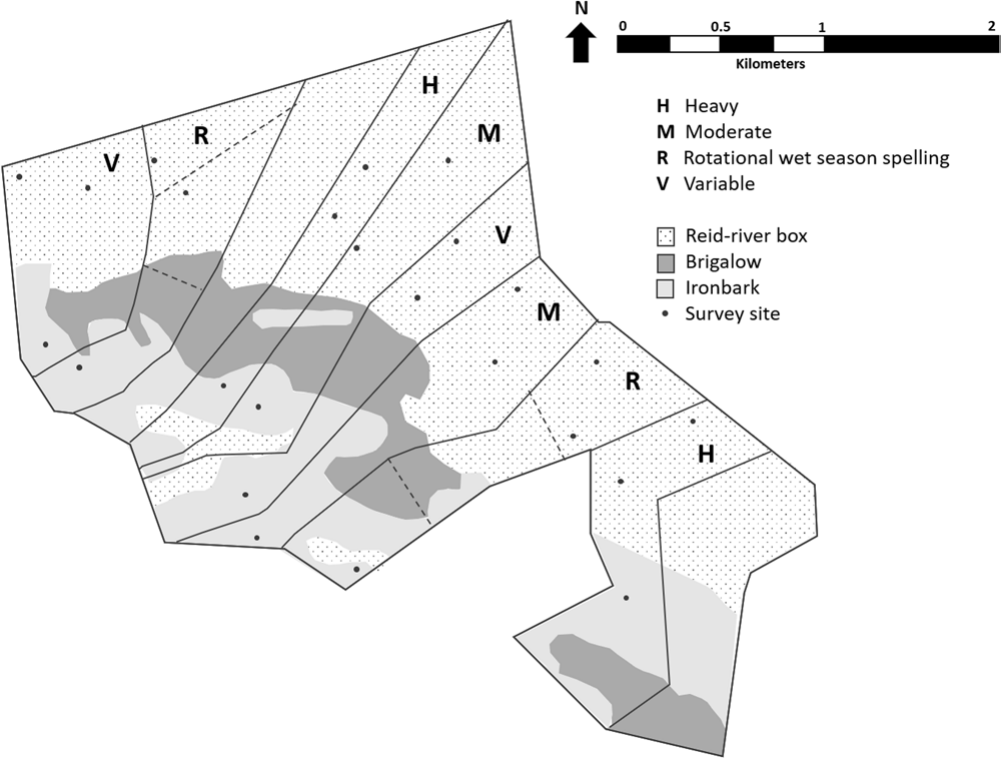
The Wambiana grazing trial. Survey sites were located in four different grazing treatments (Heavy, Moderate, Rotational wet-season spelling and Variable) and in two vegetation communities (Reid-river box and Ironbark). A third main vegetation community, characterised by dominant Brigalow, runs in a band through the grazing trial. Map created using Google Earth imagery (Map data: Google, Digital Globe), drawn in Microsoft PowerPoint 2016 by Heather Neilly.

The vegetation is open savanna woodland with two dominant communities: Reid River Box (*Eucalyptus brownii*, hereafter ‘Box’) and Silver-leaf Ironbark (*Eucalyptus melanophloia*, hereafter ‘Ironbark’). A band of Brigalow (*Acacia harpophylla*) separates the two dominant communities (Fig. 1; see^37^ for detailed floristic descriptions). Each vegetation community has an understorey of tropical grass species and patchily distributed Currant Bush (*Carissa ovata*). Each paddock contains both dominant vegetation communities.

### Mammal surveys

Twenty-four 1-ha sites (100 m × 100 m) were established, with six sites located in each of the four grazing treatments (Fig. 1). Each site was in either the Box or the Ironbark vegetation community.

Four surveys were conducted; these took place in 2014 and 2015, in both April (end of the wet season) and October (end of the dry season). Along the centre line of each 1-ha site, a rectangular, wire cage trap (710 × 305 × 305 mm) was set at 0 m, and another at 50 m. Each cage trap was baited with a ball of peanut butter, rolled oats, and vanilla essence. Cage traps were checked before dawn and closed for the remainder of the day (with bait removed), before being re-opened and re-baited in the late afternoon. Each trapping session ran for a 10-night period. Captured animals were removed from traps, identified, weighed, measured, marked with a unique ear clip combination and then released at the site of capture. Species nomenclature followed^22^. All experimental protocols were approved by the QLD Department of Environment and Resource Management under a ‘Scientific research and educational purposes permit’ in accordance with the relevant guidelines and regulations and approved by the James Cook University Animal Ethics Committee.

### Micro-habitat surveys

Micro-habitat features were measured at each site during each survey session (Table 1). Within a 1-ha site, 3 × 100 m parallel transects, 50 m apart, were established. Along the transect, ground cover was categorised as bare ground, leaf litter >5 mm, leaf litter 5–10 mm, rock, fine woody debris (<10 cm diameter) or coarse woody debris (>10 cm diameter). Vegetative cover along the transect was categorised as grass (with grass height also recorded), shrub or tree. Both ground and vegetative cover measures were converted into mean percentages. Additionally, all trees within one metre on either side of the transects were identified and their height and diameter at breast height (DBH) was measured. We also measured mean percentage canopy cover, mean distance to nearest tree (m) > 2 m tall, mean percentage canopy connectivity, mean percentage trees with hollows (further detail in Table 1).

**Table 1:** A description of the micro-habitat variables surveyed at each site.

Micro-habitat characteristics	Description
Ground cover	A tape measure was laid on the ground along a 100 m transect. The amount of bare ground (BG), rock, leaf litter (LL) and leaf litter depth (mm), fine woody debris (<10 cm diameter) (FWD), coarse woody debris (>10 cm diameter) (CWD) was recorded in cms and converted into a percentage.
Vegetative cover	Along the 100 m transect, the amount of grass (and grass height), shrub and other vegetative cover was recorded and converted into a percentage. Mean grass height was calculated for each transect.
Other features	Other ground features were measured along the 100 m transect including termite mounds (TM), and burrows.
Trees	Any tree that fell 1-m either side of the 100 m transect was identified and measured for diameter at breast height (DBH) (cm) and height category (m)
Canopy Cover (%)	Estimated canopy cover *via* spherical densiometer.
Canopy connectivity (%)	The percentage of overstory trees sampled that had overlapping canopy or branches.
Tree hollows (%)	The percentage of overstory trees sampled that had hollows or cavities visible from the ground.

### Data Analysis

A range of analyses were performed in R^38^, with specific packages cited where relevant.

### Population dynamics

Brushtail possum population dynamics for the entire grazing trial were examined by analysing our mark-recapture data in *Rcapture* ^39^. Population dynamics could not be modelled among treatments or between vegetation types as individuals could move between sites. Due to the hierarchical nature of our trapping data, we used a robust design analysis, i.e., within a trapping session (10 consecutive days of capture) the population is assumed to be closed (not experiencing immigration or mortality), but between each trapping session the population is considered open. A robust design can generate estimated abundances for each trapping session and survival rates between periods.

### Grazing treatments, vegetation type and micro-habitat use

A generalised linear mixed model, with poisson distribution, was used to analyse the response of brushtail possum abundance to grazing treatment and vegetation type (fixed effects), with year and season included in the model as random effects. To analyse the response of brushtail possum abundance to the micro-habitat variables at each site, we used a generalised linear model with negative binomial distribution (to account for overdispersion). Both analyses were performed using *lme4* ^40^. The optimal models were selected using the Akaike Information Criterion (AICc) from the *dredge* function in *MuMInI* ^41^. The optimal models were validated by examining the deviance residuals. Pairwise comparisons among grazing treatment and between vegetation type were made using the Tukey test in *lsmeans* ^42^.

Additionally, we performed habitat selection analyses on the variables in the optimal models, using *adehabitatHS* ^43^. Our data was structured as a ‘design II study’, i.e., each trapped individual was identified and habitat use was recorded for each individual. Since micro-habitat variables were collected at a site level, when an individual was trapped at a site, it was considered to be using the micro-habitat variables at that site. The habitat availability was measured at a population scale, i.e., habitat units were considered equally available to all individuals. We used Manly selection ratios^44^ to calculate habitat availability to habitat use, for each animal, for each habitat type and then averaged over all animals.

### Individual home-ranges and movement

Although not the focus of this study, home range size was calculated, in *adehabitatHR* ^43^, for individuals with at least five recaptures. First, we estimated the kernel utilisation distribution (KUD) and then extracted the 95% and 50% home range contours. KUD contours are used here to visualise individuals use of grazing treatments and vegetation types.

### Data Availability

The datasets generated during and analysed during the current study are available from the corresponding author on reasonable request.

## Results

### Population dynamics

**Figure 2:**
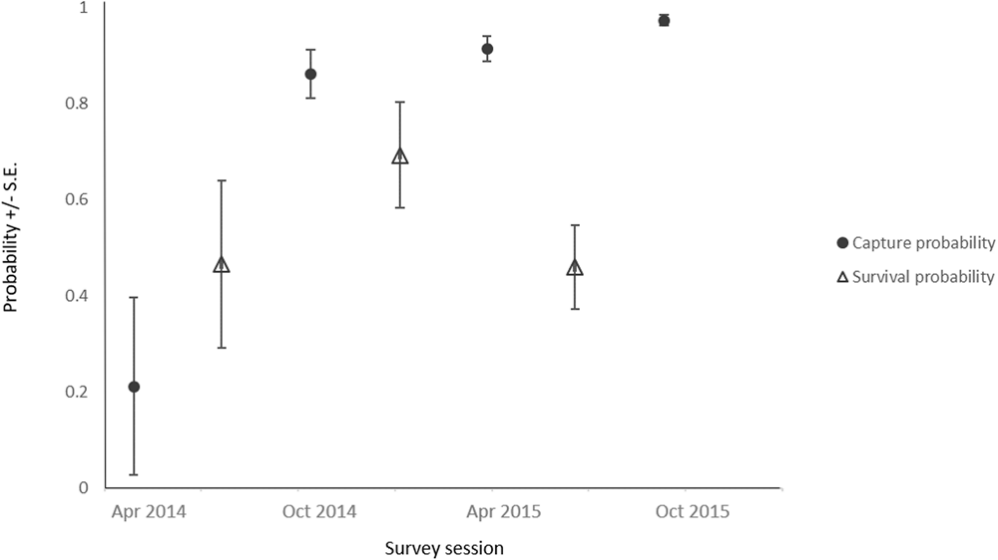
Estimated capture probability at each survey session and survival probability between survey sessions +/− S.E., for *Trichosurus vulpecula vulpecula* population.

Across four surveys and 1920 trap nights, 63 unique individuals were captured and 38 were subsequently recaptured (Table 2). Overall, it was estimated that 79 ± 21.7 (S.E.) individuals inhabited the survey area over the two year period, with high capture probabilities in the last three surveys and survival probabilities between surveys around 50% (Fig. 2). Breeding occurred multiple times per year, as unfurred joeys were recorded in all surveys.

**Table 2:** Trap success, recapture rates, sex ratios and population size of *Trichosurus vulpecula vupecula* captures at each trapping session.

Survey session	Trap nights	Trap success %	No. animals trapped	Density/ha	No. unique trapped females	No. unique trapped males	Recapture rate all %	Sex ratio F:M	Females with pouch young (%)
April 2014	480	2.50	12	0.01	5	5	0	1:1	40.0
October 2014	480	10.6	51	0.03	8	26	60.8	1:3.25	50.0
April 2015	480	19.2	92	0.05	14	33	69.6	1:2.36	35.7
October 2015	480	19.8	95	0.04	12	33	86.3	1:2.75	50.0
Overall	1920	13.0	250		17	46	72.4	1:2.71	47.1

### Grazing treatments, vegetation type and micro-habitat use

**Figure 3:**
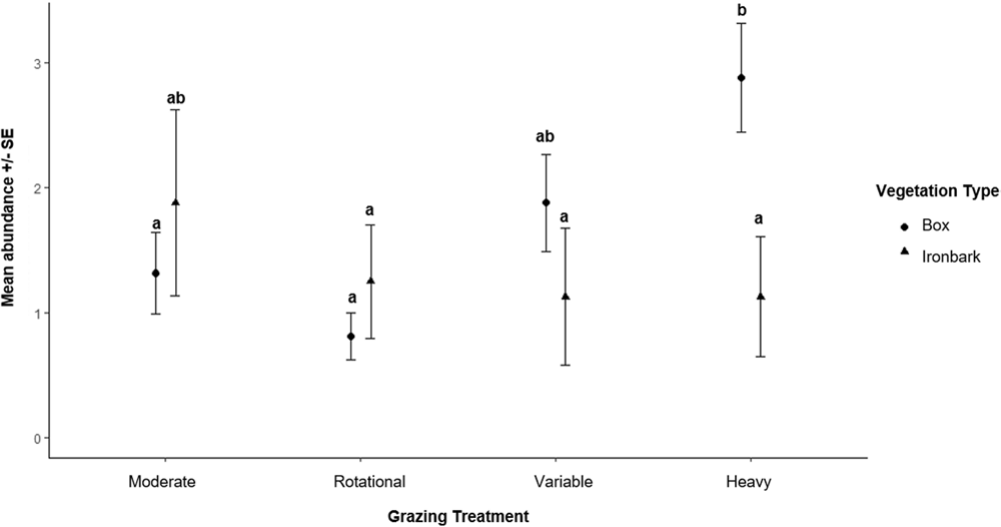
Mean *Trichosurus vulpecula vulpecula* abundance +/− SE, significantly different terms indicated by different letters (Tukey posthoc α = 0.05).

The optimal generalised linear mixed model included grazing treatment and vegetation type and the interaction between these two variables (AICc = 317.7). The next best model only contained grazing treatment, however the AICc value was sufficiently lower (ΔAICc = 2.49) to accept the more complex, optimal model. The highest mean abundance of brushtail possums was found in the Heavy grazed Box sites (Fig. 3). Within the Box vegetation community, the Heavy and Variable treatments had a significantly higher abundance than the Moderate and Rotational treatments. There was no significant difference among grazing treatments within the Ironbark vegetation community. On average, individuals selected Heavy (Manly selection ratio = 1.42 ± 0.29 SE) and Variable treatments (1.11 ± 0.21 SE) and avoided Moderate (0.95 ± 0.20 SE) and Rotational treatments (0.52 ± 0.13 SE). Of 63 individuals marked, 20 individuals used multiple grazing treatments (31.7%), and 43 only used 1 of the grazing treatments: 16 only used Heavy (25.4%), 16 only used Variable (25.4%), 5 only used Moderate (7.94%) and the remaining 6 individuals only used the Rotational treatment (9.52%). On average, individuals selected the Box vegetation type (Manly selection ratio = 1.13 ± 0.11 SE) and avoided the Ironbark vegetation community (0.74 ± 0.22 SE). Of 63 individuals marked, 4 individuals used both vegetation types (6.35%), 44 only used the Box (69.8%) and 15 only used the Ironbark vegetation type (23.8%).

**Figure 4:**
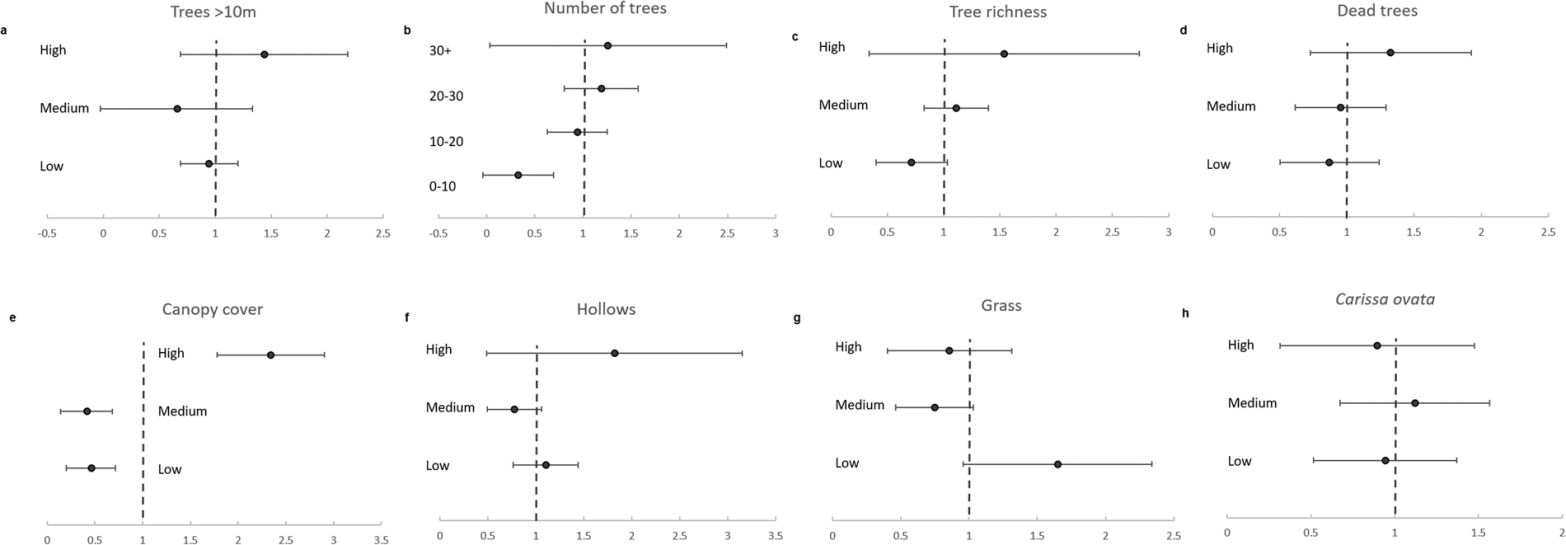
Mean Manly selection ratios with 95% confidence interval for *Trichosurus vulpecula vulpecula* use of micro-habitat variables. Micro-habitat variables are ‘selected’ where Manly selection ratio is >1, and ‘avoided’ where Manly selection ratio is <1. Results are considered significant were the 95% confidence intervals do not cross 1.

When modelling brushtail possum response to micro-habitat variables, the optimal model contained the terms Canopy Cover, *Carissa ovata* cover, Number Dead Trees, Number Trees >10 m, Tree Richness and Grass Cover (AICc 298.5, ΔAICc = 2.21). All terms were ecologically relevant to brushtail possum micro-habitat use, so these variables were used to test habitat selection using Manly selection ratios. On average, brushtail possums selected the highest category of Trees >10 m, Number of trees, Tree Richness, Canopy Cover and Hollows (mean Manly selection ration >1), however due to high variability, only the selection of high Canopy Cover was significant (Fig. 4a–h). Brushtail possums selected low Grass cover (Fig. 4g), and significantly avoided Number of Trees 0–10 (Fig. 4b) and low and medium Canopy Cover (Fig. 4e).

### Individual home-ranges and movement

**Figure 5:**
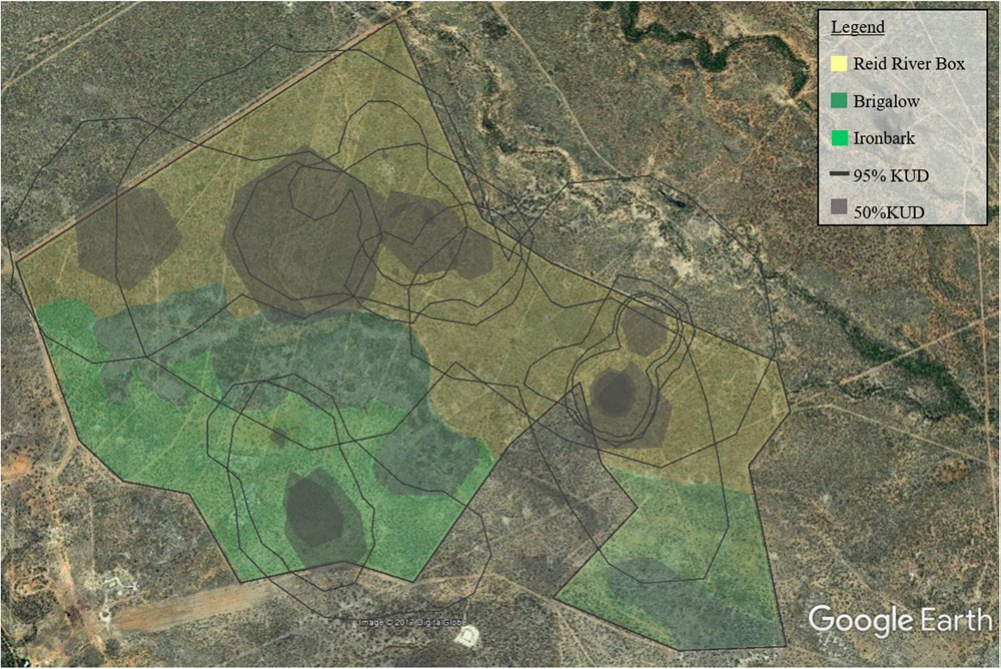
The 95% and 50% kernel utilisation densities (KUD) of *Trichosurus vulpecula vulpecula* individuals in relation to the Wambiana grazing trial vegetation communities. Map data: Google, Digital Globe.

**Figure 6:**
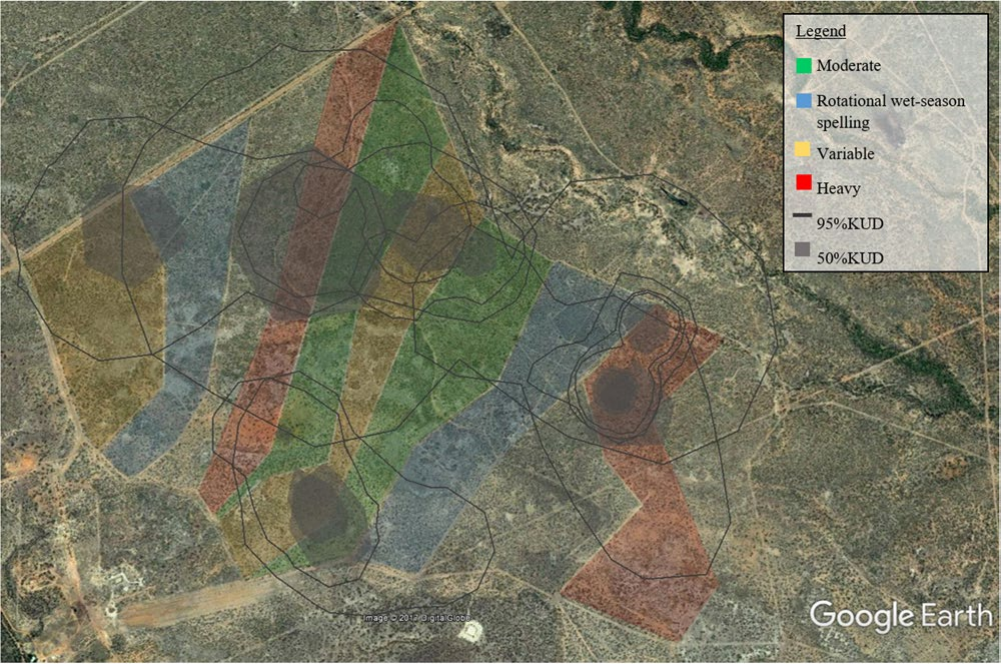
The 95% and 50% kernel utilisation densities (KUD) of *Trichosurus vulpecula vulpecula* individuals in relation to the Wambiana grazing trial grazing treatments. Map data: Google, Digital Globe.

Twelve individuals had sufficient recapture data (at least five recaptures) to calculate kernel utilization densities (KUDs). Two individuals were recaptured more than five times (ID 28 and 42), but were only ever recaptured at the same location, therefore home range analysis could not be done. One individual (7.14%), used both vegetation types, two individuals used Ironbark only (14.28%), and the remaining 11 individuals only used the Box vegetation type (78.6%) (Table 3, Fig. 5). Most individuals used more than one grazing treatment, the greatest percentage of relocations overall was in the Heavy, followed by Variable, then Moderate, and finally Rotational wet-season spelling (Table 3, Fig. 6). Home range sizes were variable; the mean 95% KUD was 181.44 ha ± 62.95 SE and the mean 50% KUD was 22.91 ha ± 7.66 SE. Individual 20 was identified as an outlier and was omitted from the mean KUD contour calculations (Table 3).

**Table 3:** The number of relocations, the vegetation type used, the percentage of relocations in four grazing treatments, the kernel utilisation densities (KUD) at 95% and 50% and the sex of *Trichosurus vulpecula vulpecula* individuals captured at least five times.

ID	No. relocations	Vegetation type used	% Relocation in each grazing treatment				95% KUD (ha)	50% KUD (ha)	Sex
			Heavy	Variable	Rotational	Moderate			
20	6	Box & Ironbark	66.6	33.3	0	0	3161.7	785.9	M
3	11	Box	81.8	0	0	18.2	762.3	92.9	F
4	16	Box	93.8	0	6.2	0	66.7	5.9	M
22	5	Box	100	0	0	0	45.9	7.2	M
23	9	Box	11.1	55.6	0	22.2	164.7	23.6	M
24	10	Box	100	0	0	0	35.6	5.2	F
26	9	Box	88.9	0	0	11.1	253.1	22.5	M
41	6	Box	16.7	33.3	50	0	268.2	37.2	M
44	15	Box	0	33.3	0	66.7	56.2	7.2	M
60	15	Box	12.5	37.5	0	50	85.6	10.6	M
42	12	Box	0	100	0	0	n/a	n/a	M
28	6	Box	0	100	0	0	n/a	n/a	M
71	16	Ironbark	12.5	37.5	0	50	93.6	13.7	M
2	11	Ironbark	0	18.2	18.2	63.6	163.9	26.1	M

## Discussion

High capture probabilities suggested our brushtail possum population estimates were reliable, despite being much lower than recorded densities in wetter areas of Australia^27,45,46^. The individuals on the grazing trial had sufficient food quantity and quality to reproduce, even in 2015, when rainfall was low and food availability would likely have been lower than in the other years of the study^29^. Brushtail possums primarily eat *Eucalyptus sp*. leaves, present in both vegetation types, but will exploit seasonal food sources during fruiting or flowering events^47^. Seasonal differences in resource use could potentially affect detectability, however, while spotlighting we never saw groups of individuals congregating to feed on specific trees (unpublished data; HN pers obs). Additionally, the use of a standardised trapping methodology minimised differences in detectability, compared with subjective survey methods such as spotlighting.

In our study, brushtail possums clearly preferred the Box vegetation. This vegetation type was characterised by a higher total number of trees and a higher tree species richness than the Ironbark. High canopy cover was also more common in the Box vegetation, which was the micro-habitat feature most strongly selected by brushtail possums. Overall, individuals selected the most complex arboreal habitat available, as has been observed in other locations where this species select rainforest and forest communities over woodland^48^. Although not tested here, the preference for Box may be linked to a preference for particular *Eucalypt* species. Brushtail possums avoid species containing certain secondary plant metabolites, particularly tannins^49^, so it is possible the Box supports preferred food sources compared to the Ironbark vegetation type.

Overwhelmingly, individuals only used one habitat type or the other. If recapture rates were very low, a chance observation of such a pattern would be more likely, but recapture rates were relatively high in this study. If you only look at the individuals captured five or more times, the exclusive use of a certain vegetation type is still evident. Sex, body weight or reproductive traits did not explain why certain individuals were using certain vegetation types. The lack of relationship between breeding status and habitat is typical of brushtail possums: reproductive success of this species is not sensitive to habitat type or disturbance, because they are highly physiologically resilient^50^. Furthermore, it is unlikely that territorial individuals were excluding others, as many of the estimated home ranges overlapped, suggesting some level of social tolerance, as has been observed elsewhere^45^. It is possible that individuals remained in a particular vegetation type because they were restricted from moving between the two habitats^51^. The band of Brigalow vegetation that divides the Box and Ironbark on our study site, may be acting as matrix habitat between two suitable habitats, affecting movement. Although this vegetation type has canopy cover comparable to the Box, tree richness is relatively low and the dominance of *Acacia harpophylla* may limit suitable food resources (HN pers obs). Indeed, the majority of individuals that used both vegetation types did so at the far south-eastern side of the trial where the Box and Ironbark intrograde directly, with no division by Brigalow. Conversely, in a highly fragmented agricultural landscape, subspecies *Trichosurus vupecula hypoleucus* have been observed moving hundreds of metres through treeless gaps between vegetation patches^52^. In this case, matrix habitat did not impede *T. v. hypoleucus* movement. *T. v. hypoleucus* are a geographically isolated subspecies of the common brushtail possum, and are smaller, have denser fur and a more omnivorous diet than other subspecies^53^. The diet and morphological differences may explain the behavioural differences between these subspecies.

In addition to a preference for specific arboreal micro-habitat features, brushtail possum individuals also preferred low percentage cover of grass, which occurred most commonly at sites in the Heavy grazed treatment. The Rotational wet-season spelling treatment has the highest grass cover, and was avoided. The preference for the Heavy treatment occurred only in the Box vegetation. Different responses to grazing in different vegetation communities has been observed in other taxa^3,17^. In this case, it was the combination of the complex arboreal micro-habitat (found in the Box), and a more open ground layer (found in the Heavy grazing treatment), that brushtail possums preferred. In this study, where trees are not cleared and frequent fire is not part of the management regime, brushtail possums are not only resistant to heavy grazing but in the Box, they prefer it.

While brushtail possums spend the majority of their time in the trees, they come to the ground to move between trees where canopy is not connected^28^. Grass cover and height is important for some species to facilitate movement through agricultural matrices^54^ and this may be the case for brushtail possums, although it is not known how much they rely on visual cues for navigation. In northern parts of Australia, low ground cover, caused by over grazing, fire, or both, increases small terrestrial mammals’ risk of predation from introduced predators^55^. The combination of low ground cover and predation has been implicated in the decline of many tropical marsupials^56^. The relatively high numbers of brushtail possums in the Heavy grazed sites and individual recaptures, suggests that it is unlikely that predation rates are increased by low ground cover in this instance. Presumably this is because an arboreal mammal spends much less time on the ground than a terrestrial mammal, and in addition, dingo and feral cats were observed in very low numbers due to sustained control efforts on and around the grazing trial (HN pers obs). Future research could deploy GPS collars to investigate the mechanism behind the brushtail possum’s preference for low grass cover, including quantifying the amount of time this species spends on the ground.

There are obvious limitations when using mark-recapture data only, as opposed to GPS tracking data, or a combination of both, to analyse habitat selection, movement and home ranges. GPS collars can track individuals’ habitat use over their entire range, instead we only have data on their habitat selection at a site level. We have, however, collected very detailed micro-habitat data at each site, and this can provide insight into habitat use at scales relevant to brushtail possums. It would likely not be feasible to collect micro-habitat data with this level of detail across an individual’s entire range. Additionally, we have collected data from 63 unique individuals over two years, which may not have been possible with a GPS tracking study, in which the cost of GPS collars can be a limiting factor to the number of individuals tracked^57^. One particular limitation of our results is the calculation of kernel utilisation densities based only on mark-recapture data. As such, we were unable to interpret home range sizes in detail, but rather, we used this analysis to help visualise the patterns of individuals’ use of the vegetation types and grazing treatments.

One benefit of using a large-scale, experimental grazing trial is that we can isolate the impact of different grazing strategies on vertebrate fauna, which can be very difficult in areas where grazing also interacts with disturbance from fire and tree clearing^4^. However, it is important to acknowledge that species’ declines in rangelands are most likely to be the result of cumulative impacts from multiple threats, including events that occurred historically^58^. The brushtail possum is an example of a species, despite being highly adaptable and resilient, has been unable to cope with pressure exerted by multiple threats including grazing. In urban environments, brushtail possums can thrive as long as there are trees or anthropogenic structure they can use^33^. Likewise, in our study the brushtail possum thrives in the Heavy grazing treatment where arboreal habitat is essentially intact. Other studies have also shown that retaining trees is more important for arboreal fauna than the grazing intensity^5,17,18^. Presumably, if heavy grazing was combined with a practice directly impacting trees (e.g., tree clearance), brushtail possums would respond differently^59^. Our study suggests that heavy grazing by itself is not impacting the brushtail possum negatively, however, it is clearly essential to consider all grazing-related disturbances holistically, when making rangeland management decisions.

This insight into habitat selection may assist with optimising reintroduction programs for the brushtail possum. Identifying locations with high tree richness, high canopy cover and low grass cover may enhance reintroduction success. Additionally, certain matrix habitats may act as movement barriers, potentially limiting access to seasonal food resources.

More generally, this study confirms that species responses to grazing are complex. The different response of the same species in two vegetation types we observed here, suggests that grazing management recommendations need not only be species-specific, but also vegetation-community-specific. A future focus on the vegetation-grazing interaction may help to identify where grazing pressure is more, or is less, impactful on fauna populations and provide important context to determine the benefits of ‘land-sharing’ versus ‘land-sparing’ decisions^59–61^.

For arboreal mammals, reptiles and birds to persist in grazed landscapes, tree retention is essential. Global rangelands vary in their extent of arboreal habitat and therefore arboreal fauna^1,4^, however it is widely accepted that tree retention generally increases species richness^3,62^. Therefore, compared to intensive agriculture (where trees are routinely cleared), extensive rangelands (where trees are retained) have the potential to support higher faunal diversity. Rangelands, under specific management, may be areas where agriculture and conservation can successfully co-exist.
